# Engineered exosomes with RNA‐motif short hairpin RNA loading and Rab4‐boosted production enable controlled ferroptosis in endometrial carcinoma

**DOI:** 10.1002/btm2.70163

**Published:** 2026-08-28

**Authors:** Jiarui Zhang, Yuwei Yao, Wan Shu, Shuangshuang Cheng, Guanglei Zhong, Jia Yu, Jinhua Chen, Kejun Dong, Yingying Peng, Jun Zhang, Hongbo Wang

**Affiliations:** ^1^ Department of Obstetrics and Gynecology, Union Hospital, Tongji Medical College Huazhong University of Science and Technology Wuhan China; ^2^ Hubei Key Laboratory of Biological Targeted Therapy, Union Hospital, Tongji Medical College Huazhong University of Science and Technology Wuhan China; ^3^ Hubei Provincial Clinical Medical Research Center for Cancer Immunotherapy Wuhan China

**Keywords:** endometrial carcinoma, ferroptosis, RNA motif, targeted therapy, therapeutic exosomes

## Abstract

Therapeutic challenges in endometrial carcinoma (EC) arise from the limited efficacy and toxicity of current treatments. Although exosome‐based RNA interference shows promise, its clinical translation is hindered by inefficient cargo loading, low yields, and poor tumor targeting. We have engineered an exosome platform (cRGD‐ExoM) that integrates the following innovations: Firstly, RNA motifs enable the enrichment of shRNA loading by over 80‐fold for targeting of ferroptosis regulators (glutathione peroxidase 4/ferroptosis suppressor protein 1/ferritin heavy chain [GPX4/FSP1/FTH]). Secondly, Rab4 silencing amplifies exosome biogenesis via dysregulated endosomal recycling, enhancing tumor cell uptake by impairing endosome maturation—a dual‐action mechanism that boosts both production and delivery. Thirdly, cRGD peptides confer αvβ3‐integrin‐specific targeting. cRGD‐ExoM induces potent ferroptosis by increasing lipid peroxidation and downregulating GPX4/FSP1/FTH, significantly suppressing EC tumor growth in vivo without causing systemic toxicity. The platform's modular design allows for spatiotemporal control of loading, production, and targeting, demonstrating its scalability. This study provides new insights into the precision treatment of endometrial cancer by developing engineered, multifunctional, exosome‐based therapeutic drugs that combine mechanism precision and translational feasibility in tumor treatment.

## Graphical abstract

We engineered a multifunctional exosome platform (cRGD‐ExoM) utilizing RNA motifs for highly enriched shRNA payload loading and Rab4 silencing for amplified exosome biomanufacturing. By targeting avβ3‐integrin and downregulating core ferroptosis regulators (glutathione peroxidase 4/ferroptosis suppressor protein 1/ferritin heavy chain [GPX4/FSP1/FTH]), cRGD‐ExoM triggers potent, tumor‐specific lipid peroxidation in endometrial carcinoma. This dual‐engineering approach effectively bridges the gap between scalable exosome production and precision‐targeted therapy.

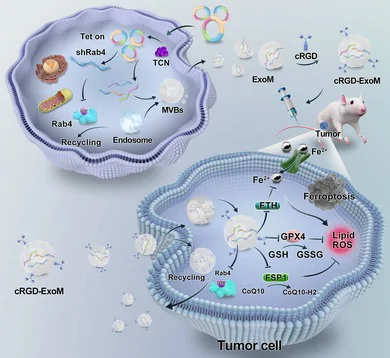

## 1 INTRODUCTION

Endometrial carcinoma (EC) is the most prevalent gynecologic malignancy in developed countries. The incidence of EC has risen over the past decade, particularly among younger women under 40.^1^ While early‐stage EC patients exhibit favorable prognoses with traditional therapies such as surgery and radiotherapy, advanced or recurrent cases remain clinically intractable due to metastasis resistance and limited therapeutic options.^2^ Current strategies, including platinum‐based chemotherapy and targeted agents (anti‐PD‐1/PD‐L1 (Programmed cell death protein 1/Programmed death‐ligand 1) antibodies), face challenges such as systemic toxicity, high costs, and narrow applicability driven by tumor heterogeneity.^3^ These challenges emphasize the critical importance of developing new therapies that can precisely target tumors, operate at the molecular level, and be delivered efficiently on a larger scale.

Extracellular vesicles (EVs), especially exosomes ranging from 30 to 150 nm, are gaining attention as effective drug delivery tools because of their natural biocompatibility, low immunogenicity, and their fundamental ability to cross biological barriers.^4^, ^5^ Notably, exosomes can encapsulate therapeutic nucleic acids (siRNA/shRNA) or proteins for target gene regulation in tumor cells.^6^ However, conventional loading methods (such as electroporation or passive co‐incubation) suffer from critical drawbacks: low load encapsulation efficiency and damage to the exosome membrane structure, which can affect their functionality in vivo.^7^, ^8^ Recent advances in understanding how mammalian cells endogenously sort and deliver RNAs via EVs provide a paradigm‐shifting solution. As comprehensively reviewed recently, the sorting of cytosolic RNAs into EVs is a highly orchestrated process governed by specific molecular mechanisms, including interactions between RNA sequence motifs and dedicated RNA‐binding proteins (RBPs).^9^ Specific RNA structural motifs have been identified to direct endogenous microRNAs (miRNAs) into exosomes via interactions with RBPs like heterogeneous nuclear ribonucleoprotein A2/B1 (hnRNPA2B1).^10^ By integrating these motifs into synthetic short hairpin RNAs (shRNAs), we hypothesized that exosome enrichment of therapeutic shRNAs could be achieved without compromising vesicle integrity and thus be used in the treatment of endometrial cancer (EC).

Another bottleneck in exosome‐based therapeutics is the scalable production and tumor‐specific delivery. Exosomes derived directly from parent cells are often not produced in sufficient quantities.^11^ Also, their inconsistent uptake by target cells can further reduce their effectiveness.^12^ The Rab GTPase family, key regulators of intracellular vesicle trafficking, presents actionable targets for biomanufacturing optimization. For example, recent studies have shown that knocking down the expression of Rab family members increases exosome secretion.^13^, ^14^, ^15^ Building on this, we propose a dual‐engineering approach: Rab4 silencing to amplify exosome production and enhance tumor cell internalization, combined with RNA motif‐driven shRNA loading for controlled payload delivery.

Ferroptosis is the iron‐dependent form of regulated cell death driven by lipid peroxidation.^16^ Our prior work revealed that EC cells develop intrinsic ferroptosis resistance through compensatory upregulation of glutathione peroxidase 4 (GPX4) and ferroptosis suppressor protein 1 (FSP1).^17^, ^18^, ^19^ Notably, while pharmacological ferroptosis inducers such as RSL3 and erastin potently triggered ferroptosis in EC cell lines in vitro, their therapeutic efficiency in vivo was limited by poor tumor accumulation and systemic toxicity, which is a challenge that emphasizes the need for tumor‐targeted delivery systems to harness ferroptosis therapeutically.^20^ Core regulators of ferroptosis—including GPX4, FSP1, and ferritin heavy chain (FTH)—are frequently overexpressed in EC tumors, rendering them resistant to conventional therapies.^21^ Silencing these targets via RNA interference (RNAi) could potentiate ferroptosis. However, systemic delivery of naked shRNAs faces rapid degradation and off‐target effects. To address this, we engineered exosomes with αvβ3‐integrin‐targeting peptides cyclic arginine‐glycine‐aspartic acid (cRGD) for tumor‐selective accumulation, leveraging the overexpression of αvβ3 integrins in EC vasculature and tumor cells.^22^

Herein, we introduced a multi‐modal exosome platform that synergistically addresses the critical challenges of RNAi delivery in EC. RNA motif‐driven engineering enabled selective enrichment of therapeutic shRNAs within exosomes by utilizing endogenous RNA sorting machinery, bypassing the inefficiencies and structural compromises of conventional loading methods. Rab4 silencing in engineered cells amplified exosome biogenesis through dysregulated endosomal recycling pathways, while parallel Rab4 knockdown in target tumor cells enhanced exosome uptake via delayed early endosome maturation—a dual strategy that concurrently optimizes production yields and target engagement. Finally, cRGD peptide functionalization conferred αvβ3‐integrin‐specific tropism, directing exosomes to tumor cells and vasculature with high precision. Collectively, this platform achieves spatiotemporal control over ferroptosis induction, demonstrating potent tumor suppression in preclinical models while preserving systemic biocompatibility. Our research proposes a new approach to using ExoM as customizable therapeutic tools, offering new insights into targeted EC therapy.

## 2 RESULTS AND DISCUSSION

### 2.1 Exosome extraction and characterization

The expression system in Figure 1a was designed and synthesized. It contains the switch component, a modified component of the Rab system, and therapeutic shRNAs that needs to be enriched in the engineered exosomes. We transfected the expression system into basal cells, specifically human umbilical vein endothelial cells (HUVECs) and switched on gene expression by adding the trigger molecule tetracycline (TCN) of the switch assembly TetR‐TetO. The engineered exosomes (herein referred to as ExoM, which are loaded with therapeutic shRNAs and Rab4 silencing modules) was collected by means of ultracentrifugation. Firstly, we verified the effect of the switching module by controlling the incorporation of TCN using systematic fluorescence expression (Figure S1). We characterized the obtained exosome ExoM by physical and chemical methods, and circular vesicles with a double‐membrane structure, with a diameter of about 100 nm, were seen under transmission electron microscopy (TEM) (Figure 1b). Further by nanoparticle tracking analysis (NTA), the specific diameter was verified and the diameter peaked at 107 nm, while in general agreement with the TEM results (Figure 1c). Exosome biogenesis originates from intracellular multivesicular bodies (MVBs).^23^ Tsg101 is a key component of the endosomal sorting complex (Endosomal Sorting Complex Required for Transport [ESCRT]), which is involved in the formation of MVBs and cargo sorting; CD63 and CD9 are members of the four‐transmembrane protein family, which are predominantly enriched at the late endosomal/MVB membranes and on the surface of exosomes, and therefore, they have been widely used as positive marker proteins for exosomes.^24^ In contrast, Calnexin is a glycoprotein folding enzyme found mainly in the endoplasmic reticulum (ER). Since the ER is not involved in exosome biogenesis, Calnexin serves as a negative marker for exosomes. Therefore, the expression of exosome‐positive markers (TSG101, CD9, and CD63) and negative markers (Calnexin) in purified exosomes and their parental cells can be detected by Western blotting to validate the purity and origin of the exosomes and to exclude the possibility of contamination by organelles such as the ER. We found significantly higher protein levels of TSG101, CD9 and CD63 in exosomes than in their parental cells, and lower expression of Calnexin in exosomes, confirming the specificity of these purified exosomes (Figure 1d). Furthermore, we verified the cellular uptake capability of the ExoM by treating Ishikawa (ISK) cells with ExoM (100 μL, 1 × 10^9^ particles/mL). As visualized under fluorescence microscopy, ISK cells successfully internalized the 1,1'‐dioctadecyl‐3,3,3',3'‐tetramethylindodicarbocyanine (DiD)‐labeled ExoM (Figure 1e).

**FIGURE 1:**
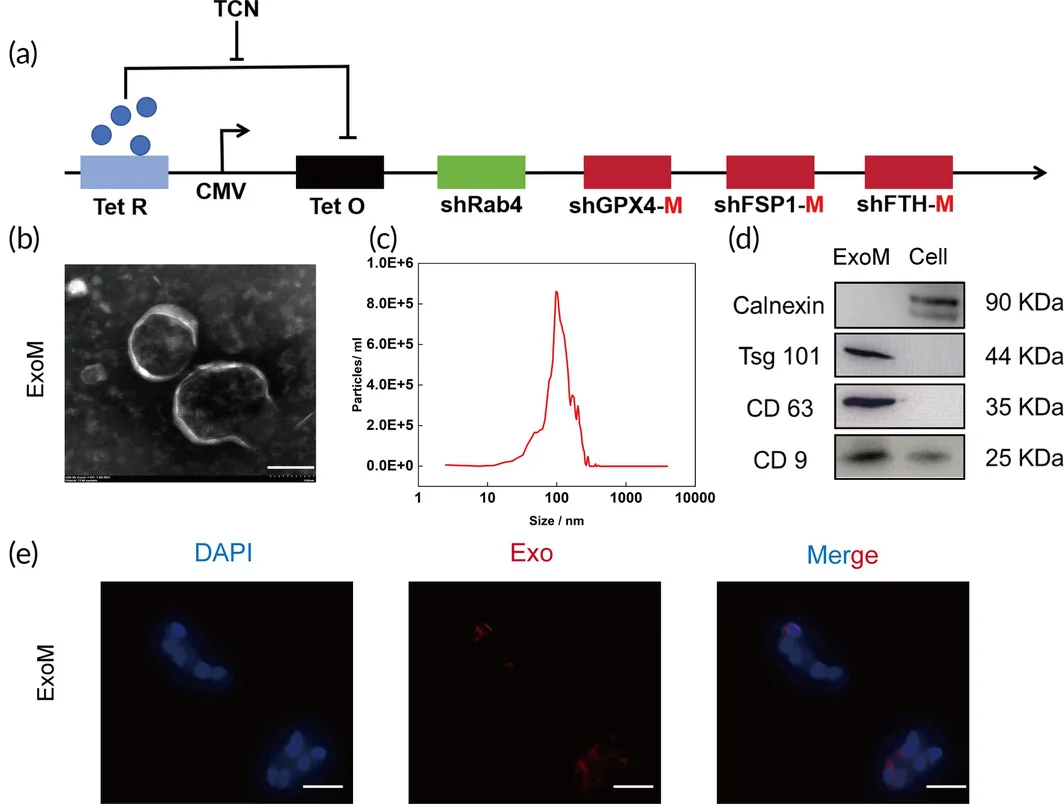
Isolation and identification of engineered exosomes (ExoM). (a) The illustration of expression system of ExoM. (b) The representative transmission electron microscopy images and mean diameters of ExoM (scale bar: 50 nm). (c) The diameter of the ExoM was confirmed by the nanoparticle tracking analysis system. (d) Western blot characterization showing the protein expression levels of exosomal markers (TSG101, CD63, and CD9) and an endoplasmic reticulum‐negative marker (Calnexin) in ExoM and parental cell lysates. Equal amounts of total protein (20 μg) were loaded per lane. (e) The uptake and internalization of DiD‐labeled ExoM was observed in Ishikawa cells (Scale bar: 200 μm). TCN, trigger molecule tetracycline.

### 2.2 RNA motif‐enabled therapeutic shRNA/miRNA enrichment

In order to verify the enrichment of RNAmotif for shRNA/miRNA, we designed shRNA/miRNA with or without motif in the expression system, respectively, and extracted exosomes after expression in basal cells and examined the expression of target RNAs in the exosomes (Figure 2a). We designed the expression system as shown in Figure 2b, selected shGPX4 and miR‐15a as enrichment targets, and verified the expression of the expression system in cells by green fluorescence of enhanced green fluorescent protein (EGFP). According to the previous literature, we designed four RNA motifs, transfected the basal cells with their relevant expression systems respectively, and extracted the exosomes in the culture supernatant after 48 h of culture.^10^ The expression levels of the target shRNA/miRNAs in cells and exosomes were detected by Reverse transcription quantitative polymerase chain reaction (RT‐qPCR). The results, as shown in Figure 2c,d, indicated that the expression of overexpressed shRNAs and miRNAs was essentially identical in the producer cells, which was consistent with the intracellular fluorescence expression (Figure S2). However, in the extracted exosomes, all four RNA motifs were able to enrich shRNA/miRNA compared to the control group. Among them, the M1 motif (CGGGAG) exhibited the most potent enrichment capacity, achieving an 81.75‐fold enrichment for shGPX4 and a 67.5‐fold enrichment for miR‐15a. The expression of all target RNAs was normalized to miR‐138‐5p, an internal reference that remained stable across different exosome samples.^25^ To further evaluate the therapeutic and translational potential of this platform, we determined the absolute loading efficiency using absolute Quantitative polymerase chain reaction (qPCR) quantification based on a standard curve (Figure S3). Consistent with the relative fold‐change data, the M1 motif mediated the highest absolute loading capacity, reaching approximately 4 × 10^4^ copies of shGPX4 (Figure 2e) and miR‐15a (Figure 2f) per 10^8^ exosomes. This robust enrichment confirms that our engineered platform can yield therapeutically viable concentrations of RNAi payloads for downstream functional delivery.

**FIGURE 2:**
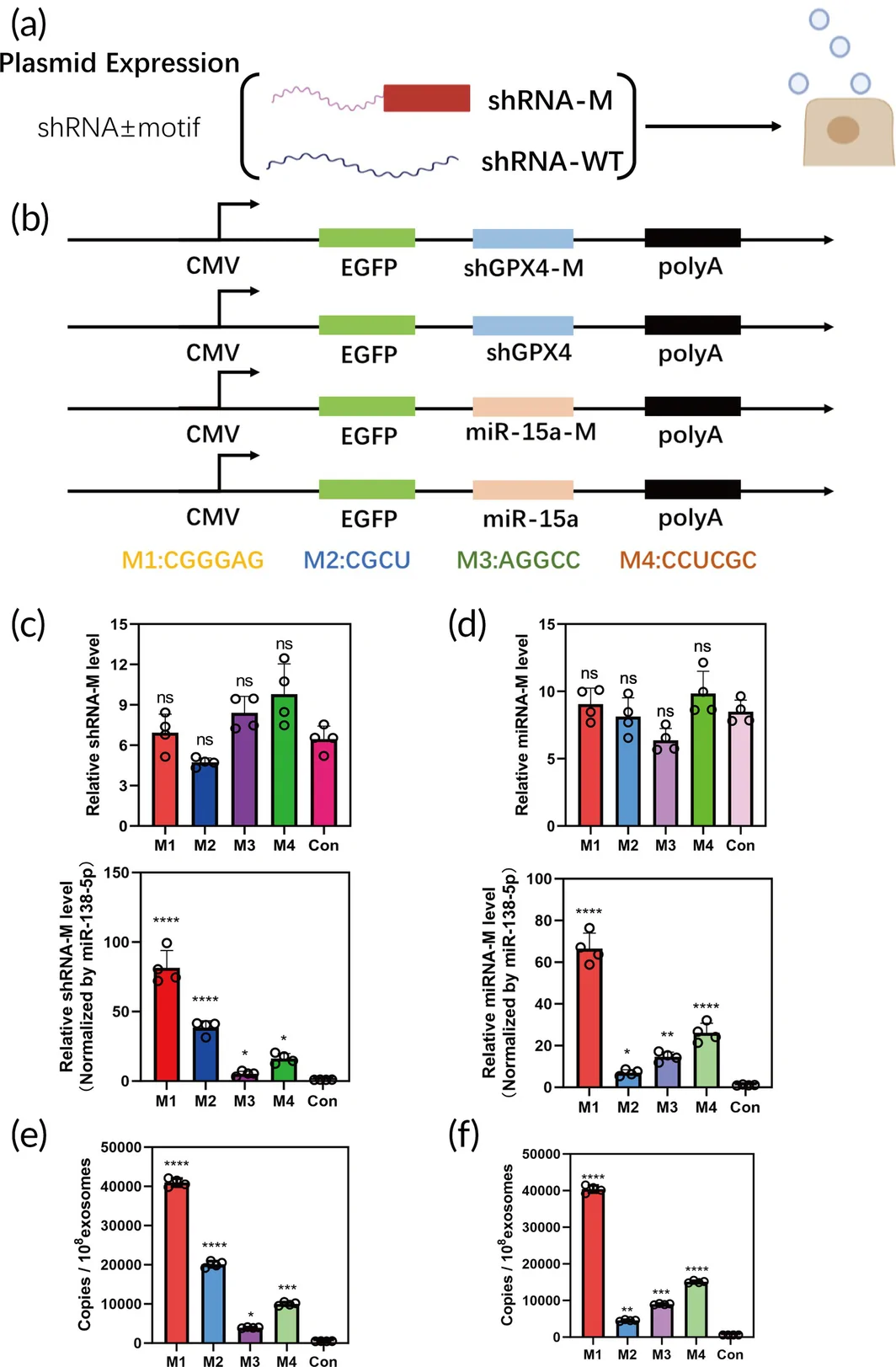
Screening and validation of RNA motifs. (a) Schematic diagram of the screening and validation process for RNA motifs. (b) Illustration of the expression system for therapeutic shRNA and miRNA with/without specific motifs. The sequences of different motifs are shown at the bottom in corresponding colors. (c) qRT‐PCR revealing the relative expression of different shGPX4‐M constructs in producer cells (top) and secreted exosomes (bottom). (d) qRT‐PCR revealing the relative expression of different miR‐15a‐M constructs in producer cells (top) and secreted exosomes (bottom). (e, f) Absolute quantification of shGPX4 (e) and miR‐15a (f) copy numbers packaged within the engineered exosomes (presented as Copies/10^8^ exosomes), demonstrating the robust payload enrichment driven by the M1 motif. (Con represents the Phosphate‐buffered saline (PBS)‐treated control group) (*p < 0.05, **p < 0.01, ***p < 0.001, and ****p < 0.0001; ns, no statistical significance). EGFP, enhanced green fluorescent protein.

### 2.3 Rab4 silencing amplifies exosome biogenesis and enhances recipient‐cell uptake via endosomal recycling reprogramming

The Rab family belongs to the small GTPases, which are key regulators of intracellular vesicular transport and are closely related to the uptake, recycling and secretion of exosomes. For example, Rab14 and Rab22a regulate cargo transport between the Golgi and early endosomes (EEs), Rab4 and Rab35 are involved in the recycling of EEs, Rab11a is involved in the long‐loop recycling of recycling endosomes, and Rab9 is involved in the transport of cargo between late endosome (LEs) and the Golgi (Figure 3a).^26^, ^27^, ^28^ We screened for the major Rab family members and examined exosome production in basal cells after knocking down these members. As shown in Figure 3b, knockdown of Rab4a resulted in the most significant enhancement of exosome production in basal cells, achieving a 1.8‐fold increase compared to the control group. Importantly, direct statistical comparisons among the different silencing groups confirmed that Rab4a knockdown was significantly superior in promoting exosome biogenesis when compared head‐to‐head against the other top‐performing candidates, including siRab35 (*p* < 0.01) and siRab14 (*p* < 0.001). We further knocked down Rab4 in basal cells and verified the knockdown effect by Western blot (Figure 3c). Hepatocyte Growth Factor‐regulated Tyrosine Kinase Substrate (HGS) is a core component of the intracellular ESCRT pathway, which plays an initiating and key regulatory role in the formation of MVB and exosome secretion. Therefore, its expression can reflect the exosome secretion ability of cells. To rigorously evaluate this, we employed both immunofluorescence and Western blot analyses. As shown in the optimized fluorescence imaging (Figure 3d,e), the HGS signal intensity in the Rab4 knockdown group was markedly higher than that in the control group. More importantly, this upregulation was definitively confirmed by Western blot quantitative analysis (Figure 3f–h), which demonstrated a highly significant increase (approximately 2.6‐fold, *p* < 0.0001) in HGS protein levels following successful Rab4 depletion. To further investigate the relationship between Rab4 knockdown and increased exosome secretion, we analyzed the number and morphology of intracellular MVBs as well as EEs/LEs by TEM. The results showed that the number of MVBs and EEs/LEs was significantly increased in the Rab4 knockdown group compared with the control group. These results suggest that knockdown of Rab4 can enhance the exosome production of cells by decreasing the recycling of early endosome and increasing the formation of MVBs, which is consistent with the findings of previous reports.^15^

**FIGURE 3:**
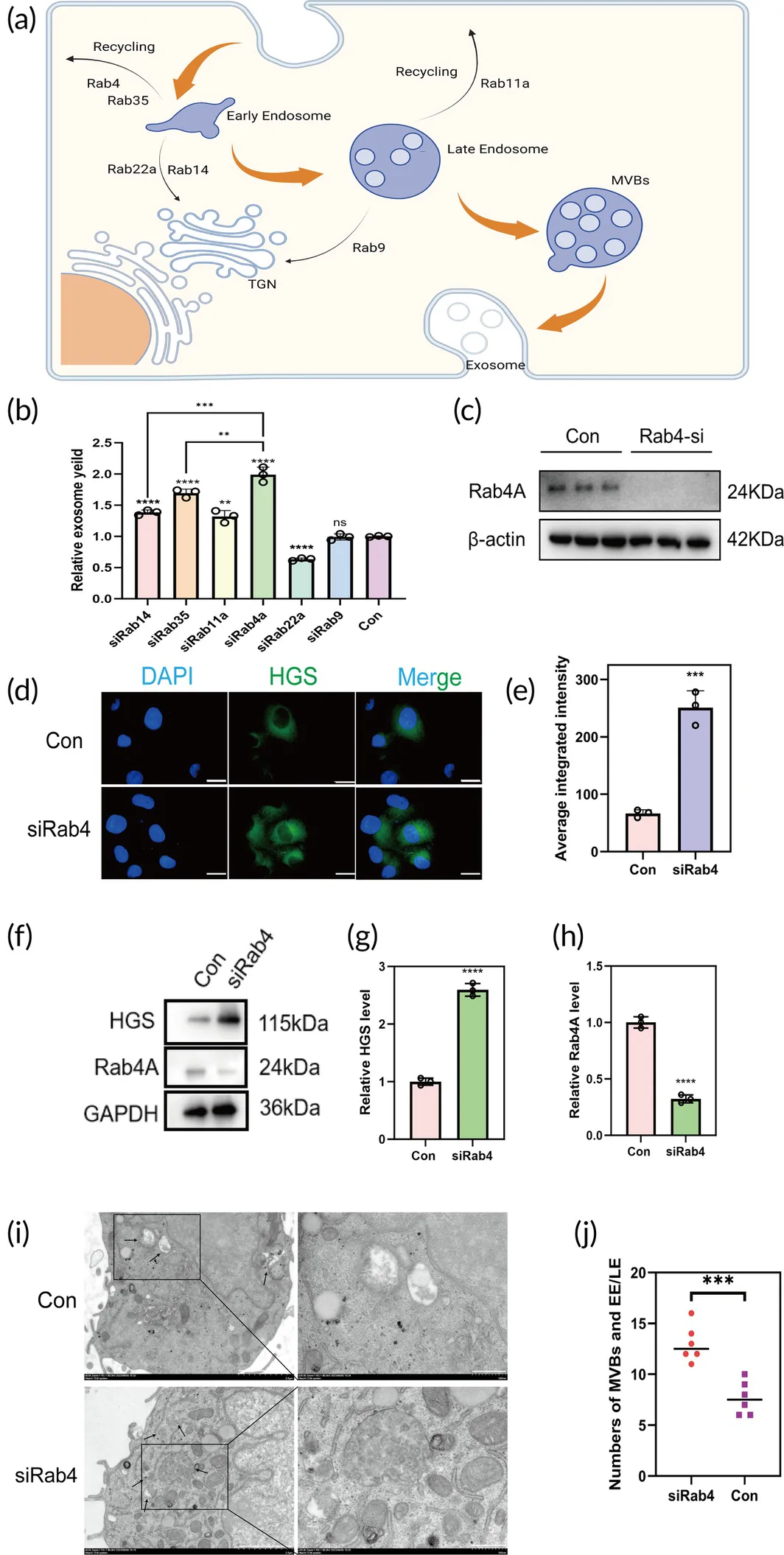
Rab4 silencing amplifies exosome biogenesis. (a) Schematic diagram of Rab family proteins involved in intracellular vesicle transport and exosome biogenesis. (b) Effects of different Rab silencing on exosome production. Human umbilical vein endothelial cells (HUVEC) cells were transfected with indicated siRNAs and the derived exosomes were calculated by nanoparticle tracking analysis (NTA). Statistical significance between the optimal siRab4a group and other key candidates (siRab35 and siRab14) was evaluated using one‐way analysis of variance with Tukey's post hoc test to confirm its superior enhancement effect (**p < 0.01, ***p < 0.001). (c) Western blot showed the knocdown of Rab4 in HUVEC cells. (d) Fluorescent microscopy analysis of the Hepatocyte Growth Factor‐regulated Tyrosine Kinase Substrate (HGS) in HUVEC cells transfected with siRab4 or siNC (scale bar: 20 μm). (e) Quantitative analysis of fluorescence intensity of HGS (***p < 0.001). (f) Western blot analysis of HGS and Rab4A protein levels in control and Rab4‐knockdown cells (g, h). Corresponding quantitative densitometry analysis of relative HGS (g) and Rab4A (h) protein levels normalized to Glyceraldehyde‐3‐phosphate dehydrogenase (GAPDH) (****p < 0.0001). (i) Representative transmission electron microscopy images showing the effects of siRab4 or siNC on exosome biogenesis in HUVEC cells. Black arrows indicate multivesicular bodies (MVBs) and early endosome (EE)/late endosome (LE) (scale bar: 2 μm, left; 500 nm, right). (j) The number of MVBs and EE/LE per cell profile. Each point represents the number of MVBs and EE/LE in each cell profile (Con represents the PBS‐treated control group) (***p < 0.001).

Rab4, as a key regulator of the rapid recycling pathway in EEs, has a dual biological function: it not only participates in the biogenesis of exosomes, but also dominates the recirculation pathway of cytosolic exosomes.^29^ Specifically, internalized exosomes can be re‐released into the extracellular matrix via Rab4‐positive recycling vesicles after sorting in EEs. Meanwhile, we found that direct intracellular overexpression of shRab4 in producer cells resulted in the packaging of shRab4 molecules into the secreted exosomes. After verifying the enrichment of shRab4 within ExoM (Figure S4), we evaluated the ability of ExoM to deplete intracellular Rab4 and enhance exosome retention in target ISK cells using fluorescence microscopy (Figure 4a). Compared with control exosomes (Exo), ExoM treatment effectively reduced Rab4 expression in recipient cells (Figure 4b) and promoted significantly greater exosome internalization (Figure 4c). The corresponding uncropped wide‐field fluorescence images used for this quantification are provided in Figure S5. To further validate the functional status of recipient cells, TEM analysis was performed, illustrating that the number of recycling endosomes was significantly reduced in ExoM‐treated cells (Figure 4d,e).

**FIGURE 4:**
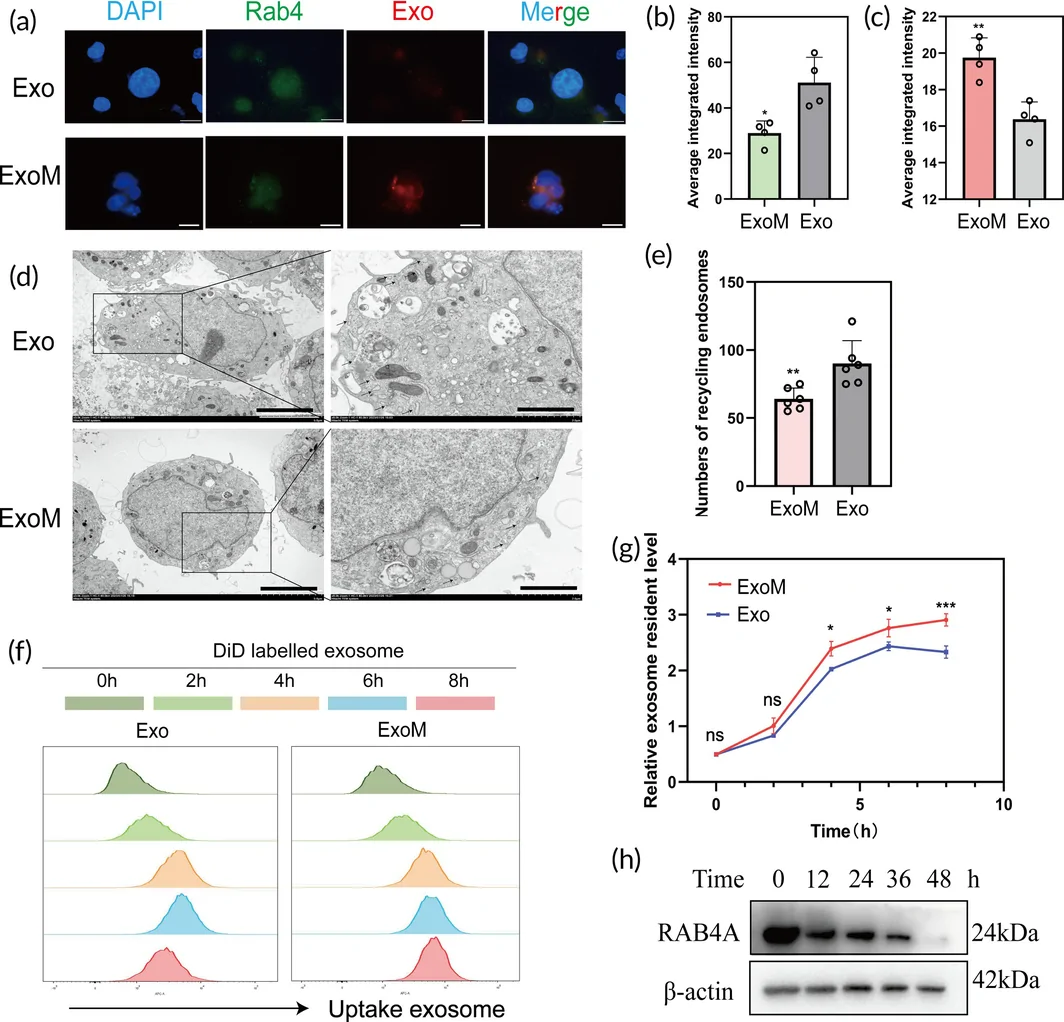
Engineered exosomes (ExoM) enhances recipient‐cell uptake via endosomal recycling reprogramming. (a) Fluorescence microscopy results show the expression of Rab4 in ISK cells and the uptake of DiD‐labeled ExoM and DiD‐labeled exosomes (Exo) (scale bar: 20 μm). (b) Quantitative analysis of fluorescence intensity of Rab4 (*p < 0.05). (c) Quantitative analysis of fluorescence intensity of DiD (**p < 0.01). (d) Representative transmission electron microscopy images showing the effects of ExoM or Exo on exosome uptake in Ishikawa (ISK) cells. Black arrows indicate recycling early endosomes (EEs) (scale bar: 2 μm, left; 500 nm, right). (e) Quantitative analysis of fluorescence intensity of recycling EEs (**p < 0.01). (f) Flow cytometry results showed the fluorescence intensity of DiD‐labeled ExoM or DiD‐labeled Exo treatment at 0, 2, 4, 6, and 8 h in ISK cells. (g) Quantitative analysis of fluorescence intensity at 0, 2, 4, 6, and 8 h in ISK cells (***p < 0.001 and *p < 0.05). (h) Time‐course Western blot analysis of RAB4A protein levels in ISK cells treated with ExoM for 0, 12, 24, 36, and 48 h, demonstrating the temporal dynamics of shRab4‐mediated knockdown.

To dynamically track exosome retention, we treated ISK cells with DiD‐labeled Exo or ExoM and monitored intracellular fluorescence over an 8‐h period using flow cytometry (Figure 4f,g). Notably, during the initial phase (0–4 h), the intracellular fluorescence of ExoM and unmodified Exo increased at nearly identical rates, indicating that basal endocytosis remains unaffected. However, beginning at approximately 4–5 h post‐incubation, a significant divergence emerged: while the intracellular fluorescence of the control Exo group plateaued and slightly decreased after 6 h—indicative of exosome recycling and efflux—the retention of ExoM continued to increase steadily up to 8 h. This delayed enhancement captures the precise biological time window required for the engineered shRab4 payload to initiate its functional blockade of the recycling pathway.

Importantly, to comprehensively address the temporal dynamics of this Rab4 feed‐forward mechanism, we performed a time‐course analysis of Rab4 protein levels in recipient ISK cells post‐ExoM treatment (Figure 4h). Consistent with the early functional divergence observed in flow cytometry, the downstream Rab4 protein levels began to noticeably decline by 12 h, with robust and near‐complete depletion sustained through 24–48 h. This precise kinetic alignment indicates that while the initial wave of endocytosed exosomes acts to “prime” the cells by delivering the RNAi payload, the subsequent exosome uptake and retention are exponentially enhanced once the Rab4 recycling machinery is structurally dismantled.

### 2.4 cRGD‐ExoM targets endometrial cancer cells in vitro and in vivo

To enhance the targeting ability of ExoM in vivo and in vitro, we modified the exosome by attaching a DSPE‐PEG2000‐cRGD peptide, which enables active tumor targeting by binding specifically to αvβ3 integrins. Using this method, we successfully produced the ExoM, which we named cyclic RGD‐modified engineered exosomes (cRGD‐ExoM) (Figure 5a). We then characterized ExoM and cRGD‐ExoM, and results of TEM showed that both exhibited a typical double‐membrane circular vesicle structure. The diameter of ExoM was approximately 100 nm, while that of cRGD‐ExoM increased to 150 nm (Figure 5b). Furthermore, NTA verified their specific particle size distributions: the ExoM diameter peaked at 107 nm and the cRGD‐ExoM diameter at 146.5 nm, in general agreement with the TEM results (Figure 5c). This increase in size is mainly attributed to the physical size of PEG2000 (20–30 nm) and the formation of its surface hydration layer in an aqueous environment. Importantly, to rule out the possibility of vesicle aggregation or fusion during the modification process, we evaluated the Polydispersity Index (PDI). Both ExoM and cRGD‐ExoM exhibited a highly monodisperse profile with PDI values well below 0.3 (Figure S6A). Furthermore, to ensure physiological reliability, we performed a serum stability assay. When incubated in medium containing 10% fetal bovine serum (FBS) at 37°C for 48 h, the hydrodynamic diameter of cRGD‐ExoM remained exceedingly stable without exhibiting massive aggregation (Figure S6B). These data confirm the robust colloidal stability of our engineered platform. To evaluate the in vitro targeting ability of cRGD‐ExoM, we co‐cultured Green fluorescent protein (GFP)‐labeled HUVEC cells (normal cell control) with ISK cells and administered 1,1'‐dioctadecyl‐3,3,3',3'‐tetramethylindodicarbocyanine (DiD)‐labeled cRGD‐ExoM and ExoM treatments, respectively. Figure 5d shows that 4',6‐diamidino‐2‐phenylindole (DAPI) staining labels the location of all cells, GFP signals indicate normal HUVEC cells and DiD channels show cellular uptake of exosomes. Whereas ExoM was uniformly distributed, cRGD‐ExoM was significantly enriched in ISK cells. Quantitative analysis (Figure 5e) showed that almost all of the DiD red signals in the cRGD‐ExoM group were localized to ISK cells. Additionally, we investigated the in vivo accumulation capacity of cRGD‐ExoM using a xenograft tumor model. DiD‐labeled ExoM and cRGD‐ExoM (100 μL, 1 × 10^11^ particles/mL) were injected intravenously into nude mice and fluorescence intensity was detected using an in vivo imaging system (IVIS) 48 h later. The results showed that the fluorescence signal in the tumor region was significantly stronger in the cRGD‐ExoM group than in the ExoM group (Figure 5f,g), indicating that cRGD‐ExoM is more effective at enriching tumors in vivo. Overall, the results of both the in vitro and in vivo experiments confirmed that cRGD‐ExoM exhibits excellent tumor‐targeting capacity, providing a reliable basis for its further application.

**FIGURE 5:**
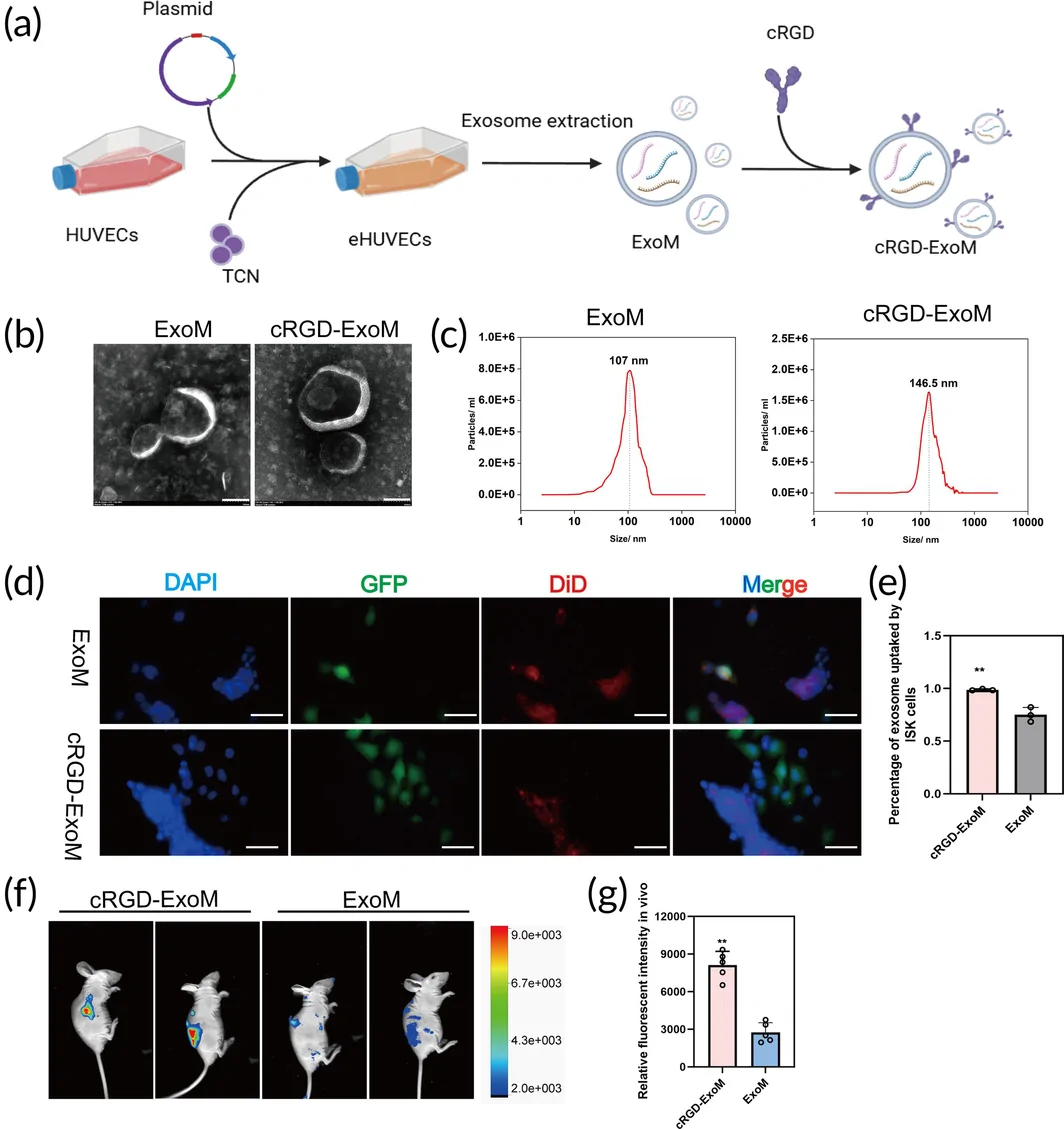
Preparation and targeting capability of cRGD‐ExoM in vitro and in vivo. (a) Schematic diagram of conjugating cRGD peptide with ExoM and generation of cRGD‐ExoM. (b) Representative transmission electron microscopy images and mean diameters of engineered exosomes (ExoM) and cRGD‐ExoM (scale bar: 50 nm). (c) The diameter of the ExoM was confirmed by the nanoparticle tracking analysis system. (d) ExoM and cRGD‐ExoM were labeled with DiD. GFP shows the location of HUVEC cells. Ishikawa (ISK) cell‐targeting ability of cRGD‐ExoM was detected in vitro (scale bar: 50 μm; **p < 0.01). (e) Quantitative analysis of exosome uptake by ISK cells. (**p < 0.01). (f, g) The tumor accumulation ability of cRGD‐ExoM was detected using a xenograft tumor model in vivo (**p < 0.01). Exo, exosomes; HUVEC, human umbilical vein endothelial cells; TCN, trigger molecule tetracycline.

### 2.5 Targeted ferroptosis induction by cRGD‐ExoM in endometrial carcinoma

To definitively confirm that cRGD‐ExoM‐induced cell death is mediated by ferroptosis, we performed a cell viability rescue assay using the specific ferroptosis inhibitor Ferrostatin‐1 (Fer‐1). As shown in Figure 6a, while cRGD‐ExoM treatment significantly reduced ISK cell viability, co‐treatment with Fer‐1 markedly rescued the cells from cRGD‐ExoM‐induced cytotoxicity. This functional evidence confirms that the observed anti‐tumor effect is primarily driven by ferroptosis. Furthermore, as shown by the subsequent Cell Counting Kit‐8 (CCK‐8) and proliferation assays (Figure 6b,c), compared with the control and Exo groups, ExoM and cRGD‐ExoM significantly reduced the cell viability of ISK cells. Plate cloning and EdU assay results also demonstrated that ExoM and cRGD‐ExoM effectively inhibited ISK cell proliferation (Figure S7). Compared with the control and Exo groups, qPCR and Western blot analysis showed that the expression of GPX4, FSP1, and FTH was significantly reduced in ISK cells treated with ExoM and cRGD‐ExoM (Figure 6d–f). Additionally, compared with the control and Exo‐treated groups, the levels of malondialdehyde (MDA) in ISK cells were significantly increased after treatment with ExoM and cRGD‐ExoM (Figure 6h). Furthermore, the levels of reactive oxygen species (ROS) in ISK cells were significantly elevated after treatment with ExoM and cRGD‐ExoM (Figure 6g,i). By measuring intracellular ferrous ions, it was found that compared with the control and Exo treatments, the levels of Fe^2+^ in ISK cells were significantly increased after treatment with ExoM and cRGD‐ExoM (Figure 6j). Additionally, ratiometric JC‐1 staining analysis indicated that ExoM and cRGD‐ExoM treatments severely disrupted mitochondrial integrity in ISK cells. The significant transition from red J‐aggregates to green monomers (a reduced Red/Green fluorescence ratio) confirmed a profound loss of mitochondrial membrane potential driven by lipid peroxidation (Figure S8). These results indicate that ExoM and cRGD‐ExoM, compared to the control and Exo, can effectively increase lipid peroxidation levels in ISK cells, thereby confirming the efficacy of ExoM and cRGD‐ExoM in inducing ferroptosis in ISK cells in vitro.

**FIGURE 6:**
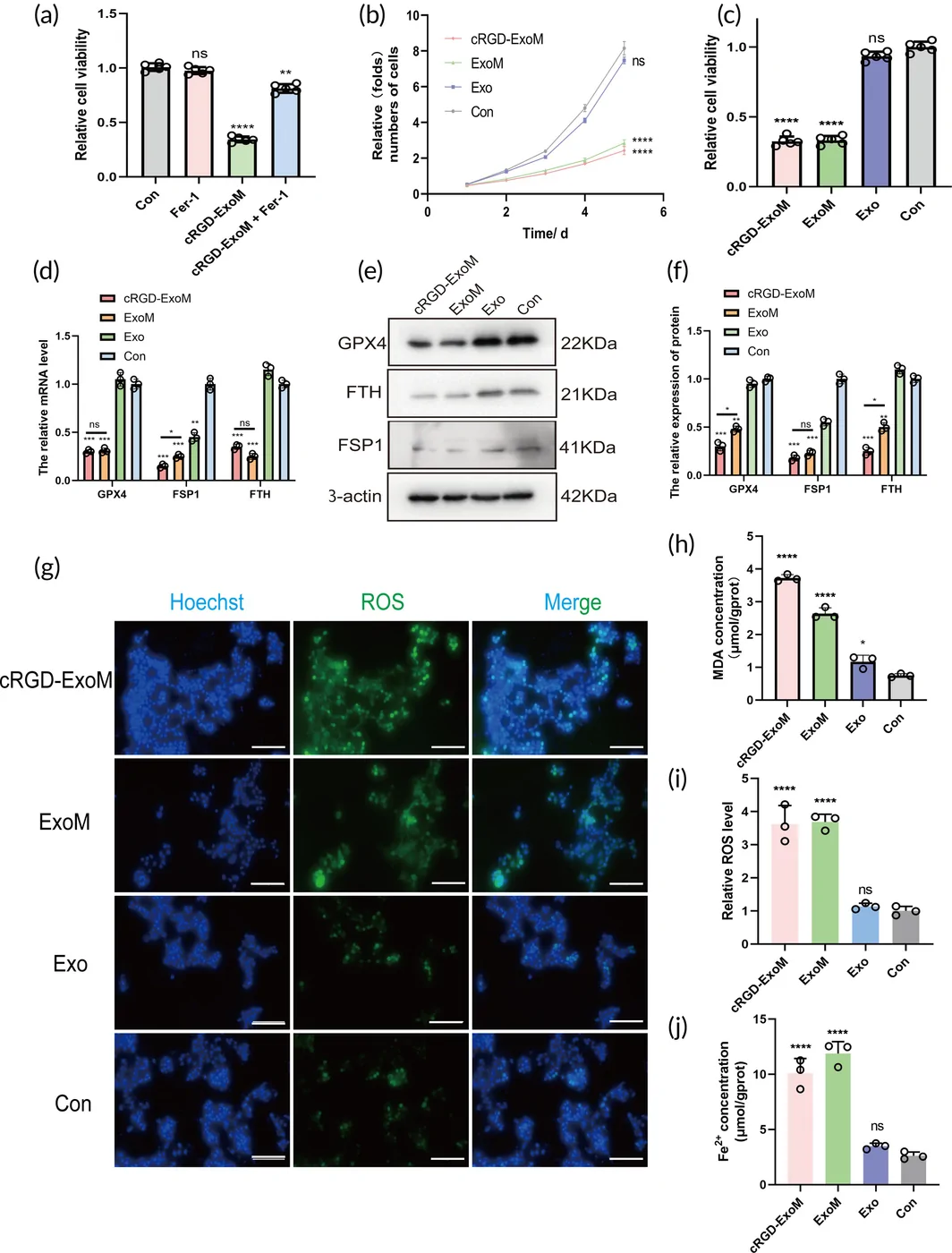
Targeted ferroptosis induction by cRGD‐ExoM in vitro. (a) CCK‐8 rescue assay showing the relative cell viability of Ishikawa (ISK) cells treated with cRGD‐ExoM in the presence or absence of the ferroptosis inhibitor Ferrostatin‐1 (Fer‐1). (b) Cell proliferation curve of ISK cells with different treatments over 5 days. (c) Cell Counting Kit‐8 assay showing the effect of different treatments on ISK cell viability at 48 h. (d) qRT‐PCR reveals the suppressive effect of cRGD‐ExoM on glutathione peroxidase 4 (GPX4), ferroptosis suppressor protein 1 (FSP1), and ferritin heavy chain (FTH) mRNA levels in ISK cells. (e, f) Western blot and corresponding quantitative analysis revealing the downregulation of GPX4, FSP1, and FTH proteins by cRGD‐ExoM. (g) Representative fluorescence images showing reactive oxygen species (ROS) accumulation (green) in ISK cells. Nuclei were stained with Hoechst (blue). Scale bar: 100 μm. (h) Malondialdehyde concentration indicating lipid peroxidation levels. (i) Quantitative analysis of relative ROS levels. (j) Fe^2+^ assay validating the accumulation of ferrous ions in ISK cells (Con represents the PBS‐treated control group) (*p < 0.05, **p < 0.01, ***p < 0.001, and ****p < 0.0001; ns, no significance). ExoM, engineered exosomes.

### 2.6 Targeted anti‐tumor therapy of cRGD‐ExoM in vivo

To investigate the therapeutic effects of cRGD‐ExoM in vivo, we established a subcutaneous xenograft tumor model using ISK cells, a well‐characterized human endometrial adenocarcinoma cell line. While orthotopic models more closely replicate the anatomical microenvironment of EC, the subcutaneous xenograft model provides a robust, highly reproducible system for the initial proof‐of‐concept evaluation of tumor growth kinetics and nanoparticle biodistribution. Furthermore, this model supports sufficient tumor neovascularization to validate the αvβ3 integrin‐targeted delivery mechanism of cRGD‐ExoM. Nude mice bearing these EC tumors were injected via the tail vein with PBS (the control group), Exo (100 μL at a concentration of 1 × 10^11^ particles per mL), ExoM (100 μL, 1 × 10^11^ particles/mL, equivalent to 85 μg/mL of exosomal protein), cRGD‐Exo‐Scr (acting as a non‐targeting RNA overload and surface modification control,100 μL, 1 × 10^11^ particles/mL, equivalent to 91 μg/mL of exosomal protein) and cRGD‐ExoM (100 μL, 1 × 10^11^ particles/mL, equivalent to 92 μg/mL of exosomal protein) on Days 3, 9, 15 and 21, after which they were euthanized on Day 25 (Figure 7a). Importantly, there was no significant difference in tumor volume and weight among the cRGD‐Exo‐Scr group, the empty Exo group, and the PBS control, explicitly indicating that neither RNA machinery overload nor cRGD surface functionalization contributed to non‐specific cytotoxicity (Figure 7b,c). Furthermore, cRGD‐ExoM treatment significantly reduced the number of Ki67‐positive cells compared to the control, Exo, and ExoM groups (Figure 7d), as well as significantly decreasing GPX4, FSP1, and FTH levels (Figure 7e). These results suggest that cRGD‐ExoM exhibits stronger targeted anti‐tumor therapeutic efficacy in vivo than the control group, Exo, and ExoM. Hematoxylin and eosin (HE) staining of the control, Exo, ExoM, and cRGD‐ExoM groups did not reveal any obvious pulmonary toxicity, cardiac damage, hepatic or renal injury, or splenic inflammatory infiltration (Figure 8a). To further rigorously validate systemic safety and rule out early‐stage subclinical functional impairment, quantitative serum biochemistry analysis was performed at the experimental endpoint. As demonstrated in Figure 8b–e, the serum levels of liver function biomarkers (alanine aminotransferase [ALT] and aspartate aminotransferase [AST]) and kidney function indexes (blood urea nitrogen [BUN] and Creatinine) in the cRGD‐ExoM‐treated mice remained entirely within normal physiological ranges. Crucially, there were no statistically significant differences (ns) among any of the groups, confirming the excellent functional biosafety of the engineered nanoplatform in vivo.

**FIGURE 7:**
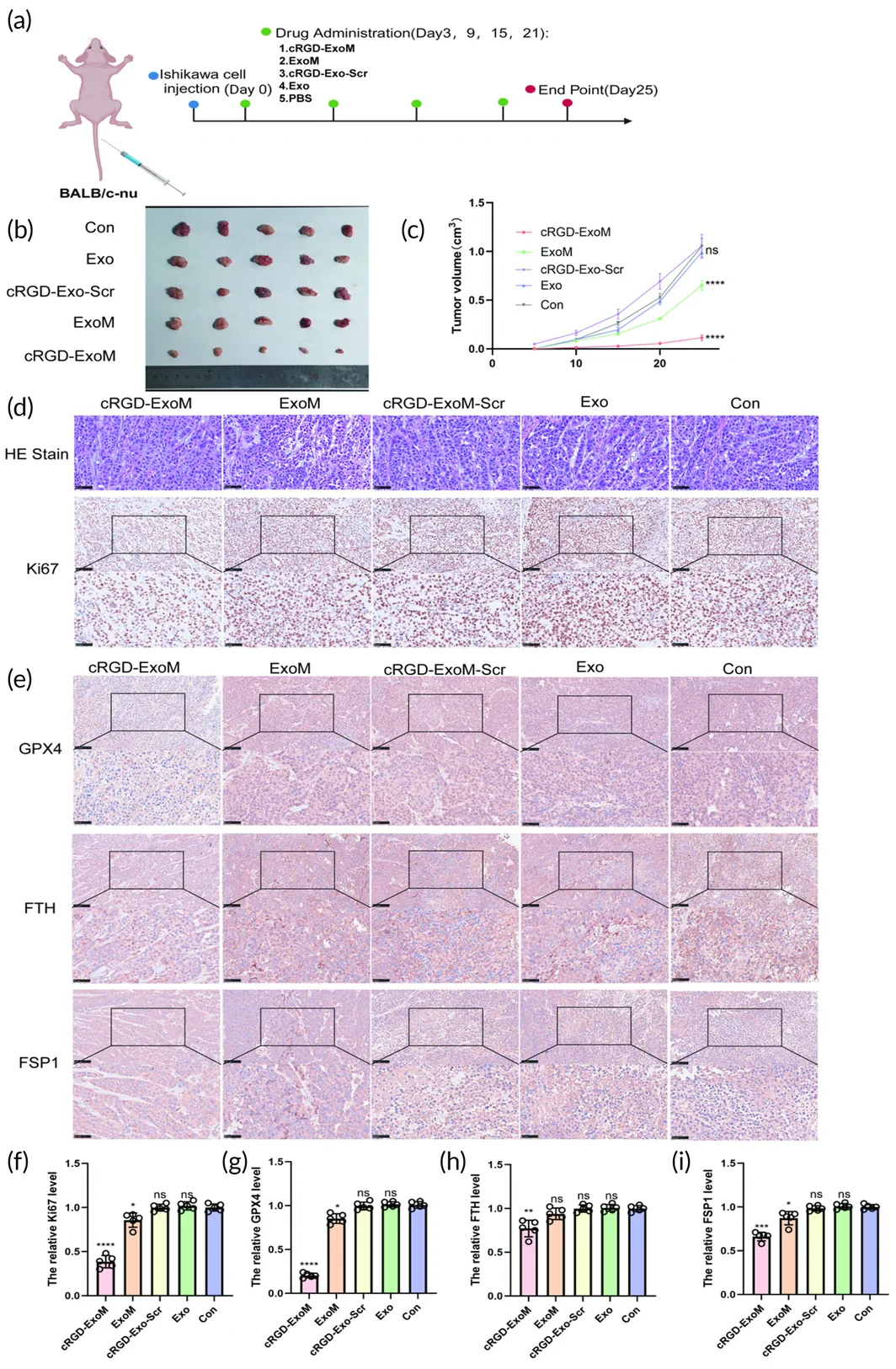
Targeted anti‐tumor therapy of cRGD‐ExoM in vivo. (a) Schematic diagram of the establishment and handling of xenograft tumor model. (b, c) The volume of subcutaneously transplanted tumors of these three groups was recorded (***p < 0.001, ****p < 0.0001). cRGD‐Exo‐Scr indicates cRGD‐modified exosomes loaded with a non‐targeting scramble shRNA sequence. (d) Hematoxylin–eosin (HE) staining and Ki67 immunohistochemistry (IHC) showed the proliferation rates of cells in endometrial carcinoma (EC) tissues (scale bar: 100 μm). (e) Glutathione peroxidase 4 (GPX4), ferritin heavy chain (FTH), and ferroptosis suppressor protein 1 (FSP1) IHC showed the expression level in EC tissues (scale bar: 100 and 20 μm). (f–i) Semi‐quantitative analysis of the relative staining intensities for Ki67 (f), GPX4 (g), FTH (h), and FSP1 (i) in tumor tissues across the four experimental groups. Data are expressed as mean ± standard deviation with individual data points displayed (*n* = 5 per group, (*p < 0.05, **p < 0.01, ***p < 0.001, ****p < 0.0001) (Con represents the PBS‐treated control group). Exo, exosomes; ExoM, engineered exosomes.

**FIGURE 8:**
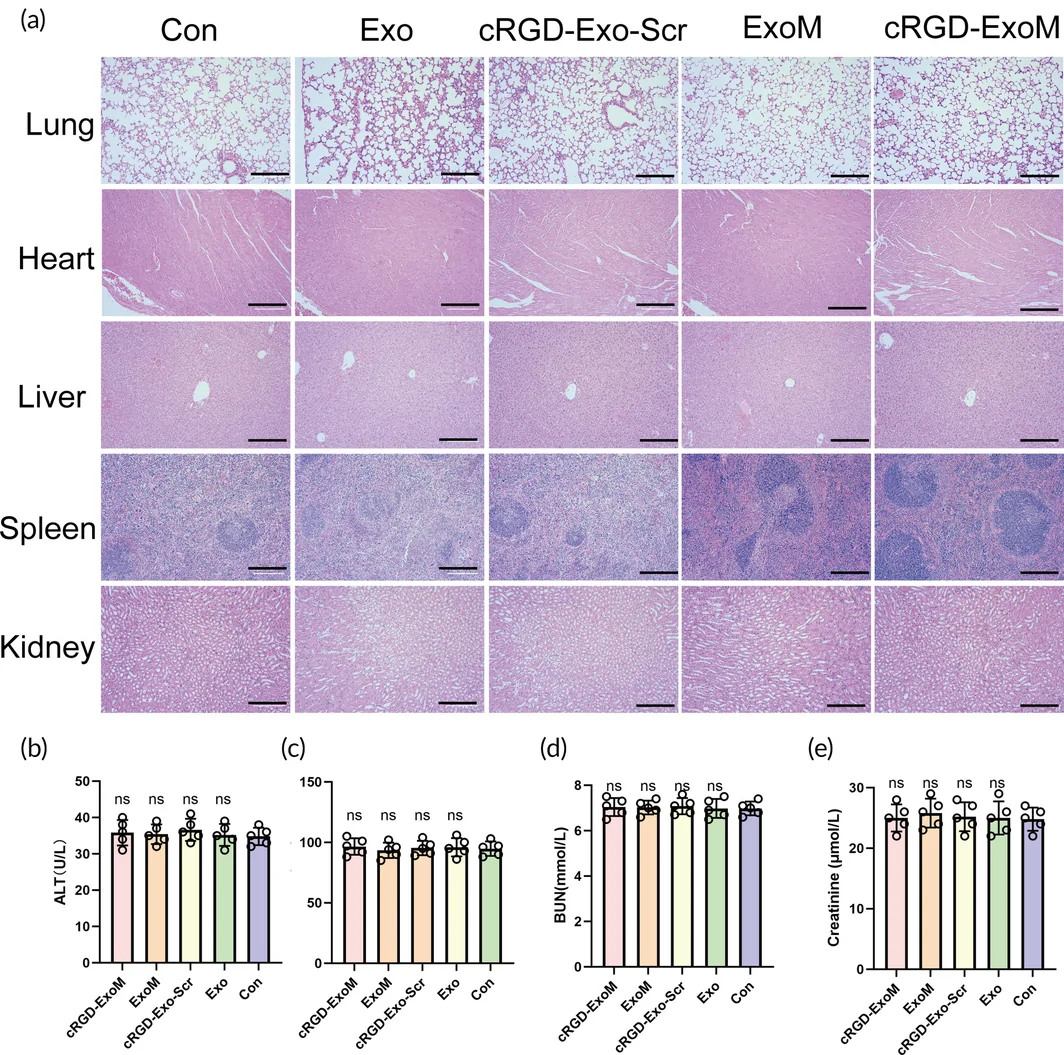
In vivo systemic biosafety and biocompatibility evaluation. (a) Representative hematoxylin–eosin staining images of major organs (lung, heart, liver, spleen, and kidney) collected from mice in different treatment groups at Day 25 post‐injection (scale bars = 100 μm). (b–e) Quantitative serum biochemical analyses including liver function parameters (alanine aminotransferase [ALT], aspartate aminotransferase) and kidney function indicators (blood urea nitrogen [BUN], creatinine). Data are presented as mean ± standard deviation with individual data points plotted (*n* = 5 mice per group; ns, no significance). Exo, exosomes; ExoM, engineered exosomes.

## 3 CONCLUSION AND DISCUSSION

EC remains an area of uneven progress. Patients with mismatch‐repair‐deficient or Microsatellite instability (MSI)‐high tumors can experience meaningful benefit from PD‐1‐based therapy, but outcomes in other molecular subtypes, especially biomarker‐negative disease, remain suboptimal. This therapeutic gap motivates delivery strategies that rewire iron handling, oxidative stress, and immune signaling within the tumor microenvironment. Against that backdrop, our data support exosome‐based RNAi as a biologically coherent and translationally plausible approach for EC, complementary to current standards of care and recent first‐line chemo‐immunotherapy advances.^30^

A mechanistic strength of exosomes is their reliance on conserved Rab GTPase networks that determine the vesicle traffic. Rab27A/B govern docking of multivesicular endosomes at the plasma membrane and control exosome release^31^; Rab11 and Rab35 regulate recycling‐endosome‐derived secretion^32^; and the Rab5 to Rab7 cascade determines endosomal maturation and degradative routing. These axes define rate‐limiting steps in exosome output and cargo fate.^33^

Within this framework, Rab4 is a central regulator of fast recycling from EEs. Altering Rab4 can affect endosome maturation and receptor recycling dynamics, which are mechanisms that could plausibly influence both exosome biogenesis and the routing of recipient cells.^34^ Our results showing increased vesicle yield after Rab4 silencing are consistent with recent biomanufacturing work demonstrating higher exosome output upon Rab4 knockdown in producer cells. We therefore posit that Rab4 modulation is a practical lever to tune both production and delivery efficiency, while acknowledging that Rab4's effects on recipient‐cell uptake need further dissection across EC models. Furthermore, while our transient shRNA delivery successfully demonstrated the truncation of the recycling pathway, future studies employing stable Rab4 genetic knockout and rescue cell lines will be valuable to provide ultimate genetic validation of this endosomal routing mechanism. Our data also resolve the kinetic paradox of exosome‐mediated Rab4 silencing. Because ExoM delivers pre‐processed mature RNAs directly to the recipient cytoplasm, the onset of Rab4 downregulation occurs within 12–24 h. This establishes a temporal “priming” effect: the initial fraction of exosomes that escapes recycling initiates Rab4 degradation, creating a feed‐forward loop that dramatically amplifies the intracellular retention of continuous in vitro exposure or subsequent in vivo therapeutic doses (e.g., Days 9, 15, and 21).

Directional release is also shaped by cell polarity. In polarized epithelia, the Rab39‐UACA‐BORC axis regulates basolateral exosome secretion, which may be relevant to the organization of endometrial tissue and the delivery of directional cargo.^14^ In parallel, the fusion step that liberates exosomes has been better defined: a syntaxin‐4/SNAP‐23/VAMP‐7 SNARE complex mediates MVB‐plasma membrane fusion in several tumor cell types without disrupting MVB biogenesis.^23^ Together, these pathways provide specific points at which to adjust vesicle output and localization.

Cargo selection can be rationally encoded. Work defining “miRNA sequence codes” shows that specific nucleotide motifs bias small RNAs toward exosomal export versus cellular retention, offering a design language for enriching therapeutic RNA cargo.^10^ Conversely, endocytosis can suppress vesicular secretion of canonical exosome‐marker proteins, highlighting membrane‐traffic constraints that intersect with cargo composition.^35^ These insights align with our observation that sequence features and trafficking state jointly shape the final vesicle payload.

The convergence of exosome engineering and Rab‐mediated trafficking control provides a foundation for next‐generation RNA‐based therapeutics in EC. Our study demonstrates that rational modulation of Rab4‐dependent endosomal recycling amplifies both the quantity and functional efficiency of therapeutic exosomes, while RNA motif‐guided shRNA enrichment and αvβ3‐integrin targeting enhance payload precision. This integrative design transforms exosomes from passive delivery vesicles into programmable nanobioreactors capable of spatially confined, temporally tunable ferroptosis induction.

Biologically, this architecture builds directly upon our group's long‐standing mechanistic investigations into ferroptosis regulation in EC. Over the past several years, we have systematically dissected multiple resistance pathways that converge on iron metabolism and lipid peroxidation control. Our previous studies established that KIAA1429 (VIRMA)‐mediated m6A modification of DDIT3 enhances ferroptosis resistance by dampening ER‐stress signaling^17^; that RAB17 suppresses Transferrin receptor (TFRC)‐dependent iron uptake and limits ferroptotic sensitivity^18^; and that the circRAPGEF5‐RBFOX2 axis modulates TFRC alternative splicing to maintain iron homeostasis and protect EC cells from ferroptotic death.^19^ These findings provide a robust mechanistic foundation for the present work. By integrating these prior insights into an exosome delivery platform, our system translates vesicle trafficking modulation into precise ferroptosis control. Enhanced intracellular exposure of shRNAs targeting GPX4, FSP1, and FTH1 dismantles key antioxidant defenses and sensitizes EC cells to iron‐dependent lipid peroxidation. In this context, the current strategy does not arise in isolation but rather extends a validated mechanistic trajectory developed by our team. Consistent with our mechanistic hypothesis, the rescue of cell viability by Fer‐1 confirms that the cytotoxicity of cRGD‐ExoM is indeed ferroptosis‐dependent, effectively translating our prior molecular insights into a therapeutic reality.

Looking forward, several avenues could accelerate translation. Mapping the broader Rab interaction network (including Rab11, Rab27, and Rab35) may reveal new regulatory nodes to fine‐tune secretion and uptake. Multi‐omics profiling of ferroptotic signatures after cRGD‐ExoM treatment could clarify downstream immunometabolic remodeling and identify biomarkers for therapeutic response. In parallel, combining exosome‐induced ferroptosis with immune checkpoint blockade or metabolic modulators may yield synergistic anti‐tumor effects, particularly in EC subtypes that exhibit limited immunotherapy responsiveness.

From a translational standpoint, scalability and safety remain pivotal considerations. Advances in bioreactor‐based exosome production, potency assays, and quantitative RNA tracking are essential to ensure reproducibility and clarity of pharmacokinetics. Furthermore, investigating autologous or universal donor‐derived exosome sources could mitigate immunogenicity further. Beyond targeted gene silencing, engineered EVs have recently demonstrated tremendous potential as next‐generation vaccine platforms for combating cancers and infectious diseases by integrating modular loading strategies.^36^ The versatile design of our ExoM platform—combining Rab‐boosted production with targeted delivery—could thus provide a broadly applicable architectural foundation for developing novel EV‐based cancer vaccines and immunotherapeutics. Together, these efforts will bridge the gap between mechanistic innovation and clinical application.

In summary, our findings propose a paradigm shift in ferroptosis‐targeted therapy leveraging Rab‐controlled exosome dynamics not only to deliver gene silencers but also to actively remodel vesicle biogenesis, uptake, and metabolic fate within tumors. This conceptual advance underscores how understanding intracellular trafficking at a molecular level can directly inform therapeutic design, offering a roadmap toward precision exosome nanomedicine for EC.

## 4 EXPERIMENTAL SECTION

### 4.1 Cell culture and treatment

Human cell line ISK (female, *Homo sapiens*, EC tissue, RRID:CVCL_D199) and normal HUVEC (female, *H. sapiens*, umbilical vein, RRID:CVCL_2959) were purchased from the Cell Bank of the Chinese Academy of Sciences (Shanghai, China) in March 2022.

The identity of ISK cells was confirmed by short tandem repeat (STR) analysis (Figure S9), and the purity of HUVEC cells was verified by von Willebrand factor (vWF) immunofluorescence staining (Figure S10). Both cell lines were confirmed to be free of mycoplasma contamination before use, as tested using a Polymerase chain reaction (PCR)‐based Mycoplasma Detection Kit (Lonza, Switzerland).

Cells were cultured in Dulbecco's Modified Eagle Medium (DMEM)/F12 (for ISK) or Roswell Park Memorial Institute (RPMI)‐1640 (for HUVEC) medium supplemented with 10% FBS (Gibco, USA) at 37°C in an atmosphere containing 5% CO_2_.

All plasmids were synthesized by GeneChem (Shanghai, China). Transfection of plasmids was performed to overexpress therapeutic shRNAs in HUVEC cells using a DNA transfection reagent (Sangon, China) following the manufacturer's protocol. An empty plasmid cytomegalovirus DNA (pcDNA) vector served as the negative control. After 48 h of transfection, fluorescence images of GFP‐expressing cells were captured using a fluorescence microscope (Olympus Corporation, Tokyo, Japan). Cell culture supernatants were collected 48 h post‐transfection for subsequent exosome extraction.

### 4.2 Exosome isolation and characterization

HUVEC cells were grown to 80% confluency and subsequently cultured in medium containing 10% exosome‐depleted FBS (System Biosciences, USA) at 37°C for another 48 h. The conditioned medium was collected and subjected to sequential centrifugation at 4°C to remove cells and large vesicles (300 × *g* for 10 min, 2000 × *g* for 20 min, and 10,000 × *g* for 30 min) using a Thermo Scientific Sorvall ST16R centrifuge. Exosomes were then isolated by ultracentrifugation at 100,000 × *g* for 70 min at 4°C using an Optima L‐100XP ultracentrifuge (Beckman Coulter, USA). The pellets were washed once in sterile PBS and centrifuged again at 100,000 × *g* for 70 min to obtain purified exosomes.

The morphology of exosomes was examined by TEM (H‐7000, Hitachi, Japan), and particle size distribution and concentration were analyzed using NTA on a ZetaView PMX‐120 (Particle Metrix, Germany) equipped with a 488 nm laser. The NTA software parameters were set to a shutter speed of 100 and a sensitivity of 80. Western blot was performed to evaluate the expression of exosomal marker proteins, including Tsg101, CD63, and CD9.

### 4.3 qRT‐PCR assay

We performed real‐time PCR analyses using SYBR Premix Ex Taq (Vazyme, China) according to the protocol. GAPDH and miR‐138‐5p were respectively used as the controls for shRNA‐M and GPX4/FSP1/FTH mRNA, and data were collected based on the comparative cycle threshold (CT) (2^−ΔΔCT^) method. All the primers were synthesized by Sangon Biotech. Specific primer sequences are listed as follows: 5′‐CCGGGTGGATGAAGATCCAACCCAA‐3′ (forward), 5′‐GAATTCAAAAACTCCCGCTGGATGAAGAT‐3′ (reverse) for shGPX4‐M1, 5′‐CCGGGCAACATCTTCGAGATCAAGA‐3′ (forward), 5′‐GAATTCAAAAACTCCCGCAACATCTTCGAG‐3′ (reverse) for shFSP1‐M1, 5′‐CCGGGCTGGAGGTGAAGAACTT‐3′ (forward), 5′‐GAATTCAAAAACTCCCGGCTGGAGGTG‐3′ (reverse) for shFTH‐M1, 5′‐AGCUGGUGUUGUGAAUCAGGCCG‐3′ (forward), 5′‐GTGCCGGTGCAGAGGT‐3′ (reverse) for miR‐138‐5p, 5′‐UAGCAGCACAUAAUGGUUUGUG‐3′ (forward), 5′‐GTGCCGGTGCAGAGGT‐3′ (reverse) for miR‐15a.

For absolute quantification of shRNA and miRNA, synthetic RNA oligonucleotides (Sangon Biotech, China) were used to generate standard curves. The synthetic RNAs were serially diluted (10^0^ to 10^8^ copies/μL) to establish the linear relationship between the log‐transformed copy number and the CT value (Figure S3). The absolute copy numbers of target RNAs in exosomes were calculated based on the standard curves and normalized to the number of exosomes (presented as Copies/10^8^ exosomes).

### 4.4 Western blot assay

For the time‐course analysis of Rab4A protein levels, ISK cells were treated with ExoM and harvested at the designated time points (0, 12, 24, 36, and 48 h). Total protein was extracted using Radioimmunoprecipitation assay (RIPA) lysis buffer and rigorously quantified using a BCA Protein Assay Kit (Beyotime Biotechnology, China). Equal amounts of protein lysates (20 μg per lane) were resolved by 10% Sodium dodecyl sulfate‐polyacrylamide gel electrophoresis (SDS‐PAGE) and subsequently transferred onto 0.22 μm polyvinylidene difluoride (PVDF) membranes (Millipore, USA). After blocking, the membranes were incubated with specific primary antibodies at 4°C for 18 h. The membranes were then washed and incubated with horseradish peroxidase (HRP)‐conjugated secondary antibodies (Abclonal, China) at 37°C for 1 h. Finally, the immunoreactive bands were visualized using an ECL Kit (Thermo Fisher, USA) and captured by a Bio‐Rad ChemiDoc MP Imaging System, with exposure times ranging from 10 s to 2 min optimized for each target protein. The primary antibodies against Rab4A, HGS, and other targets were purchased from Proteintech (China), Abclonal (China), and CST (USA).

### 4.5 Exosome uptake by ISK cells

Exosomes were fluorescently labeled using a DiD membrane dye (Beyotime Biotechnology, China) with strict normalization of background intensity. Briefly, a standardized formulation of 1 × 10^11^ particles exosome particles was incubated with a final concentration of 1 μM DiD at 37°C for 20 min in the dark. To completely eliminate any confounding background from unreacted free dye, the mixture was subjected to a rigorous wash cycle by adding 10 mL of sterile PBS followed by ultracentrifugation at 100,000 × *g* for 70 min at 4°C. The labeled exosome pellets were resuspended and their absolute concentrations re‐verified via NTA to guarantee dosing parity (1 × 10^9^ particles/mL). To examine the cellular uptake, ISK cells or HUVECs were cultured with the labeled exosomes at 37°C for 12 h. The cells were then fixed with 4% paraformaldehyde for 15 min and counterstained with 1 μg/mL DAPI (Beyotime) for 10 min to visualize cell nuclei. Cellular uptake signals were examined using a high‐fidelity fluorescence microscope (Olympus IX73) using optimized exposure settings maintained strictly constant across all experimental groups, with excitation/emission wavelengths adjusted to 644/665 nm for DiD and 358/461 nm for DAPI.

### 4.6 Co‐localization of Rab4 with uptake exosomes

The initial cultivation of ISK cells with/without Rab4 knockdown was performed on cell coverslips. Following the administration of ExoM treatment with DiD, the cells were subjected to a 12‐h culture period under standard conditions (37°C, 5% CO_2_). Thereafter, the cell coverslips were fixed and labeled with Rab4 using an immunofluorescence kit (Abkine, China). Finally, DAPI staining was performed and observed using fluorescence microscopy.

### 4.7 Verification of rapid endosomal circulation by flow cytometry

ExoM labeled with DiD (100 μL, 1 × 10^9^ particles/mL) was incubated with ISK cells with/without Rab4 knockdown. Following treatment, cells were collected at 0, 2, 4, and 8 h, and intracellular fluorescence was detected by flow cytometry using the Allophycocyanin (APC) channel (excitation 633 nm, emission 660/20 nm filter) to detect DiD signals.

### 4.8 Preparation of cRGD‐ExoM

Tumor‐targeted peptides were obtained by dissolving phospholipid (DSPE)‐PEG2000‐cRGDyK (Ruixi, China) in an aqueous buffer. Then, 50 nmol of Tumor‐targeted peptide were mixed with 1 mL (1 × 10^11^ particles) ExoM suspension and incubated overnight at 4°C, resulting in incorporation of cRGD into the ExoM. The structural purity of the DSPE‐PEG2000‐cRGDyK conjugate was verified by ^1^H‐NMR spectroscopy (Figure S11). By using size‐exclusion chromatography (qEV original columns, Izon Science), the mixture was purified to obtain cRGD‐ExoM. For stability evaluation, the PDI of the exosomes was recorded using dynamic light scattering. To assess physiological stability, purified cRGD‐ExoM was suspended in PBS supplemented with 10% FBS and incubated at 37°C. Aliquots were collected at 0, 12, 24, and 48 h, and the hydrodynamic size was continuously monitored to evaluate potential aggregation.

### 4.9 In vitro and in vivo biodistribution of exosomes

The study protocol was approved by the Committee on the Ethics of Animal Experiments of Huazhong University of Science and Technology (2022IACUC Number: 3356). Exosomes were labeled using a DiD fluorescent membrane dye. To examine the uptake and internalization of exosomes by ISK cells specifically, ISK cells were co‐cultured with the HUVEC with GFP expression at 37°C; the cells were fixed and stained with DAPI after treatment with ExoM/cRGD‐ExoM for 12 h. The cellular uptake of exosomes was observed using a fluorescence microscope. As for in vivo biodistribution, nude mice with ISK cell planted were intravenously injected with 100 μL DiD‐labeled ExoM/cRGD‐ExoM (100 μL, 1 × 10^11^ particles/mL). The IVIS (Perkin‐Elmer, USA) was utilized to test fluorescence images of mice after 48 h.

### 4.10 Cell viability assay

Cell viability was assessed by using the CCK‐8 assay (Beyotime Biotechnology, China). ISK cells (2 × 10^3^ cells/well) with treatment were seeded onto 96‐well plates with 100 μL of DMEM medium per well. Then, the medium was replaced with 90 μL of fresh DMEM medium and 10 μL of CCK‐8 solution. After 1–4 h of incubation at 37°C, the absorbance was recorded at 450 nm. For the ferroptosis rescue assay, ISK cells were pre‐treated with or without 1 μM Fer‐1 (Sigma‐Aldrich, USA) for 2 h before the administration of exosomes. Cell viability was then assessed 48 h post‐treatment using the CCK‐8 assay as described above.

### 4.11 MDA assay

MDA content was quantified using a Thiobarbituric Acid Reactive Substances (TBARS) Assay Kit (Beyotime Biotechnology, China). Cell lysates (1 × 10^6^ cells) were centrifuged at 12,000 × *g* for 10 min at 4°C. One hundred microliters supernatant was mixed with 100 μL Thiobarbituric acid (TBA) reagent and incubated at 95°C for 60 min. After cooling on ice, samples were centrifuged (1000 g, 5 min) and the absorbance of the supernatant was measured at 532 nm.

### 4.12 Intracellular ROS measurement

ROS levels were detected using the 2′,7′‐dichlorofluorescin diacetate (DCFH‐DA) probe (Beyotime Biotechnology, China). Cells were incubated with PBS, Exo, ExoM, and cRGD‐ExoM (100 μL, 1 × 10^9^ particles/mL) for 24 h, then incubated with 10 μM DCFH‐DA in 500 μL of serum‐free medium at 37°C for 20 min in the dark. Cells were washed three times with serum‐free medium to adequately remove the background probe. After washing, cells were observed using a fluorescence microscope (Excitation ~488 nm, Emission ~525 nm).

### 4.13 Detection of labile iron pool (Fe^2+^)

Intracellular Fe^2+^ was measured using the FerroOrange probe (Beyotime Biotechnology, China) according to the manufacturer's protocol. Cells were incubated with PBS, Exo, ExoM, and cRGD‐ExoM (100 μL, 1 × 10^9^ particles/mL) for 24 h. Then, cells were loaded with 500 μL of 1 μM FerroOrange working solution (diluted in serum‐free medium). After incubation at 37°C for 30 min, the cells were washed three times with Hank's Balanced Salt Solution (HBSS) and imaged by confocal microscopy (Excitation: 543 nm, Emission: 580 nm).

### 4.14 Mitochondrial membrane potential (JC‐1) assay

The mitochondrial membrane potential (ΔΨ_m_) was assessed using the ratiometric fluorescent probe JC‐1 (Beyotime Biotechnology, China). ISK cells were seeded in multi‐well plates and treated with PBS, Exo, ExoM, or cRGD‐ExoM for 24 h. Following treatment, the cells were washed with PBS and incubated with the JC‐1 working solution (10 μg/mL) at 37°C for 20 min in the dark. Cell nuclei were counterstained with Hoechst 33342. The cells were then washed twice with cold JC‐1 staining buffer to remove background dye and immediately imaged using a fluorescence microscope. In healthy cells with high ΔΨ_m_, JC‐1 forms J‐aggregates exhibiting intense red fluorescence (Excitation/Emission: 525/590 nm). In apoptotic or ferroptotic cells with depolarized mitochondria, JC‐1 remains in the monomeric form, exhibiting green fluorescence (Excitation/Emission: 490/530 nm). For quantitative single‐cell analysis, ImageJ software was used to calculate the Mean Fluorescence Intensity (MFI) of the red and green channels specifically within the cell boundaries defined by Hoechst staining. The relative mitochondrial membrane potential was expressed as the ratio of red to green fluorescence intensity.

### 4.15 Anti‐cancer efficacy study with in vivo subcutaneous xenograft tumor model

The study protocol was approved by the Committee on the Ethics of Animal Experiments of Huazhong University of Science and Technology. Nude mice (BALB/c, female, 4‐week‐old) were injected subcutaneously with 5 × 10^6^ ISK cells. Tumor volumes were calculated with the length (*a*) and the width (*b*): volume (mm^3^) = *ab* ^2^/2. All Mice were randomly divided into different groups with corresponding treatments or as controls. The animals were sacrificed, and tumor tissues were fixed, paraffin‐embedded, and sectioned. For hematoxylin–eosin (H&E) staining, sections were deparaffinized in xylene, rehydrated through graded ethanols, stained with hematoxylin solution for 5 min, rinsed, counterstained with eosin for 1 min, dehydrated, and mounted. For immunohistochemistry (IHC), tissue sections underwent antigen retrieval in citrate buffer (pH 6.0) using microwave heating. Endogenous peroxidase was blocked with 3% H_2_O_2_. Sections were blocked with 5% Bovine serum albumin (BSA) and incubated overnight at 4°C with primary antibodies against Ki67, GPX4, FSP1, and FTH. After washing, HRP‐conjugated secondary antibodies were applied for 1 h at room temperature, followed by color development using a 3,3'‐Diaminobenzidine (DAB) substrate kit and hematoxylin counterstaining. For semi‐quantitative analysis of IHC staining, representative high‐power fields were captured per section, and the Mean Optical Density (MOD) or positive staining area percentage was determined using ImageJ software (version 1.53, NIH, USA) via the IHC Profiler plugin. Statistical significance was verified via one‐way analysis of variance (ANOVA).

### 4.16 Serum biochemistry analysis

At the endpoint of the in vivo therapeutic experiments, whole blood samples were collected from the mice via cardiac puncture or retro‐orbital sampling. The blood was allowed to clot at room temperature for 2 h and then centrifuged at 3000 rpm for 15 min at 4°C to isolate the serum. The serum levels of ALT, AST, BUN, and creatinine were quantitatively determined using an automated biochemical analyzer (or commercial assay kits according to the manufacturer's protocols) to evaluate hepatic and renal functions.

### 4.17 Statistical analysis

All in vitro experiments were performed using at least three independent biological replicates (*n* ≥ 3), and in vivo animal studies utilized five biologically independent mice per group (*n* = 5). All data are presented as the mean ± standard deviation (SD) with individual data points displayed in the corresponding bar charts. Statistical analyses were performed using GraphPad 6.0 statistical software (GraphPad Software Inc., San Diego, CA). Two‐tailed student's *t*‐test was used to examine significant differences between two groups, while one‐way ANOVA was performed to assess the statistical significance between three or more groups. A *p*‐value of <0.05 was considered to indicate a statistically significant result (*p < 0.05 and **p < 0.01).

## AUTHOR CONTRIBUTIONS

**Jiarui Zhang**: Conceptualization, validation, writing—original draft. **Yuwei Yao**: Methodology, validation, writing—original draft. **Wan Shu**: Methodology. **Shuangshuang Cheng**: Investigation. **Guanglei Zhong**: Data curation. **Jia Yu**: Data curation. **Jinhua Chen**: Validation. **Kejun Dong**: Conceptualization, investigation. **Yingying Peng**: Visualization. **Jun Zhang**: Writing—review and editing. **Hongbo Wang**: Conceptualization, funding acquisition, resources, supervision, writing—review and editing.

## CONFLICT OF INTEREST STATEMENT

The authors declare no conflict of interest.

## Supporting information


**Data S1.** Supporting Information.

## Data Availability

The raw data supporting the conclusions of this article will be made available by the authors on reasonable request.

## References

[1] Gu BX , Shang XG , Yan MQ , et al. Variations in incidence and mortality rates of endometrial cancer at the global, regional, and national levels, 1990‐2019. Gynecol Oncol. 2021;161(2):573‐580. doi:10.1016/j.ygyno.2021.01.036 33551200

[2] Oaknin A , Bosse TJ , Creutzberg CL , et al. Endometrial cancer: ESMO clinical practice guideline for diagnosis, treatment and follow‐up. Ann Oncol. 2022;33(9):860‐877. doi:10.1016/j.annonc.2022.05.009 35690222

[3] O'Malley DM , Bariani GM , Cassier PA , et al. Pembrolizumab in patients with microsatellite instability‐high advanced endometrial cancer: results from the KEYNOTE‐158 study. J Clin Oncol. 2022;40(7):752‐761. doi:10.1200/jco.21.01874 PMC8887941 34990208

[4] Kimiz‐Gebologlu I , Oncel SS . Exosomes: large‐scale production, isolation, drug loading efficiency, and biodistribution and uptake. J Control Release. 2022;347:533‐543. doi:10.1016/j.jconrel.2022.05.027 35597405

[5] Yang Q , Li SS , Ou HB , et al. Exosome‐based delivery strategies for tumor therapy: an update on modification, loading, and clinical application. J Nanobiotechnol. 2024;22(1):41. doi:10.1186/s12951-024-02298-7 PMC10823703 38281957

[6] Yang BW , Chen Y , Shi JL . Exosome biochemistry and advanced nanotechnology for next‐generation theranostic platforms. Adv Mater. 2019;31(2):1802896. doi:10.1002/adma.201802896 30126052

[7] Chen MK , Chen ZX , Cai MP , Chen H , Chen ZF , Zhao SC . Engineered extracellular vesicles: a new approach for targeted therapy of tumors and overcoming drug resistance. Cancer Commun. 2024;44(2):205‐225. doi:10.1002/cac2.12518 PMC10876209 38155418

[8] Li JL , Wang JC , Chen ZG . Emerging role of exosomes in cancer therapy: progress and challenges. Mol Cancer. 2025;24(1):13. doi:10.1186/s12943-024-02215-4 PMC11727182 39806451

[9] O'Brien K , Breyne K , Ughetto S , Laurent LC , Breakefield XO . RNA delivery by extracellular vesicles in mammalian cells and its applications. Nat Rev Mol Cell Biol. 2020;21(10):585‐606. doi:10.1038/s41580-020-0251-y PMC7249041 32457507

[10] Garcia‐Martin R , Wang GX , Brandao BB , et al. MicroRNA sequence codes for small extracellular vesicle release and cellular retention. Nature. 2022;601(7893):446‐451. doi:10.1038/s41586-021-04234-3 PMC9035265 34937935

[11] Akbari A , Nazari‐Khanamiri F , Ahmadi M , Shoaran M , Rezaie J . Engineered exosomes for tumor‐targeted drug delivery: a focus on genetic and chemical functionalization. Pharmaceutics. 2023;15(1):66. doi:10.3390/pharmaceutics15010066 PMC9865907 36678695

[12] Wang CL , Xu MC , Fan QZ , Li CH , Zhou XY . Therapeutic potential of exosome‐based personalized delivery platform in chronic inflammatory diseases. Asian J Pharm Sci. 2023;18(1):100772. doi:10.1016/j.ajps.2022.100772 PMC9989662 36896446

[13] Zhao YG , Codogno P , Zhang H . Machinery, regulation and pathophysiological implications of autophagosome maturation. Nat Rev Mol Cell Biol. 2021;22(11):733‐750. doi:10.1038/s41580-021-00392-4 PMC8300085 34302147

[14] Matsui T , Sakamaki Y , Nakashima S , Fukuda M . Rab39 and its effector UACA regulate basolateral exosome release from polarized epithelial cells. Cell Rep. 2022;39(9):110875. doi:10.1016/j.celrep.2022.110875 35649370

[15] Zhang RX , Bu T , Cao RD , et al. An optimized exosome production strategy for enhanced yield while without sacrificing cargo loading efficiency. J Nanobiotechnol. 2022;20(1):463. doi:10.1186/s12951-022-01668-3 PMC9618217 36309712

[16] Chen FQ , Kang R , Tang DL , Liu J . Ferroptosis: principles and significance in health and disease. J Hematol Oncol. 2024;17(1):41. doi:10.1186/s13045-024-01564-3 PMC11157757 38844964

[17] Shen XY , Shu W , Zhang J , et al. IL‐6/KIAA1429 promotes ferroptosis resistance in endometrial cancer through m6A modification of DDIT3. Cell Signal. 2025;134:111906. doi:10.1016/j.cellsig.2025.111906 40436288

[18] Zhou X , Nie MM , Xin XY , et al. RAB17 promotes endometrial cancer progression by inhibiting TFRC‐dependent ferroptosis. Cell Death Dis. 2024;15(9):655. doi:10.1038/s41419-024-07013-w PMC11379720 39242574

[19] Zhang J , Chen SJ , Wei ST , et al. CircRAPGEF5 interacts with RBFOX2 to confer ferroptosis resistance by modulating alternative splicing of TFRC in endometrial cancer. Redox Biol. 2022;57:102,493. doi:10.1016/j.redox.2022.102493 PMC9526237 36182807

[20] Du YX , Guo Z . Recent progress in ferroptosis: inducers and inhibitors. Cell Death Discov. 2022;8(1):501. doi:10.1038/s41420-022-01297-7 PMC9800531 36581640

[21] Wu JF , Zhang L , Wu SQ , Liu Z . Ferroptosis: opportunities and challenges in treating endometrial cancer. Front Mol Biosci. 2022;9:929832. doi:10.3389/fmolb.2022.929832 PMC9284435 35847989

[22] Xiao Q , Zhao W , Wu CT , et al. Lemon‐derived extracellular vesicles nanodrugs enable to efficiently overcome cancer multidrug resistance by endocytosis‐triggered energy dissipation and energy production reduction. Adv Sci. 2022;9(20):2105274. doi:10.1002/advs.202105274 PMC9284146 35187842

[23] Liu CQ , Liu DX , Wang S , Gan L , Yang XL , Ma C . Identification of the SNARE complex that mediates the fusion of multivesicular bodies with the plasma membrane in exosome secretion. J Extracell Vesicles. 2023;12(9):e12356. doi:10.1002/jev2.12356 PMC10497535 37700095

[24] Ai YW , Guo CX , Garcia‐Contreras M , et al. Endocytosis blocks the vesicular secretion of exosome marker proteins. Sci Adv. 2024;10(19):eadi9156. doi:10.1126/sciadv.adi9156 PMC11078179 38718108

[25] Xie XD , Cheng P , Hu LC , et al. Bone‐targeting engineered small extracellular vesicles carrying anti‐miR‐6359‐CGGGAGC prevent valproic acid‐induced bone loss. Signal Transduct Target Ther. 2024;9(1):24. doi:10.1038/s41392-023-01726-8 PMC10800355 38246920

[26] Proikas‐Cezanne T , Gaugel A , Frickey T , Nordheim A . Rab14 is part of the early endosomal clathrin‐coated TGN microdomain. FEBS Lett. 2006;580(22):5241‐5246. doi:10.1016/j.febslet.2006.08.053 16962593

[27] Baek S , Chang JW , Yoo SM , et al. TMEM9 activates Rab9‐dependent alternative autophagy through interaction with Beclin1. Cell Mol Life Sci. 2024;81(1):322. doi:10.1007/s00018-024-05366-1 PMC11335249 39078420

[28] Gurung S , Perocheau D , Touramanidou L , Baruteau J . The exosome journey: from biogenesis to uptake and intracellular signalling. Cell Commun Signal. 2021;19(1):47. doi:10.1186/s12964-021-00730-1 PMC8063428 33892745

[29] Huang NC , Winans T , Wyman B , et al. Rab4A‐directed endosome traffic shapes pro‐inflammatory mitochondrial metabolism in T cells via mitophagy, CD98 expression, and kynurenine‐sensitive mTOR activation. Nat Commun. 2024;15(1):2598. doi:10.1038/s41467-024-46441-2 PMC10960037 38519468

[30] Mirza MR , Chase DM , Slomovitz BM , et al. Dostarlimab for primary advanced or recurrent endometrial cancer. N Engl J Med. 2023;388(23):2145‐2158. doi:10.1056/NEJMoa2216334 36972026

[31] Ostrowski M , Carmo NB , Krumeich S , et al. Rab27a and Rab27b control different steps of the exosome secretion pathway. Nat Cell Biol. 2010;12(1):19‐30. doi:10.1038/ncb2000 19966785

[32] Stenmark H . Rab GTPases as coordinators of vesicle traffic. Nat Rev Mol Cell Biol. 2009;10(8):513‐525. doi:10.1038/nrm2728 19603039

[33] Zhang J , Jiang Z , Shi A . Rab GTPases: the principal players in crafting the regulatory landscape of endosomal trafficking. Comput Struct Biotechnol J. 2022;20:4464‐4472. doi:10.1016/j.csbj.2022.08.016 PMC9418685 36051867

[34] McCaffrey MW , Bielli A , Cantalupo G , et al. Rab4 affects both recycling and degradative endosomal trafficking. FEBS Lett. 2001;495(1):21‐30. doi:10.1016/S0014-5793(01)02359-6 11322941

[35] Fordjour FK , Guo C , Ai Y , Daaboul GG , Gould SJ . A shared, stochastic pathway mediates exosome protein budding along plasma and endosome membranes. J Biol Chem. 2022;298(10):102394. doi:10.1016/j.jbc.2022.102394 PMC9512851 35988652

[36] Lu M , Xing H , Zhao X , Huang Y , Zheng A , Liang X‐J . Engineered extracellular vesicles as a next‐generation vaccine platform. Matter. 2024;7(12):4180‐4205. doi:10.1016/j.matt.2024.09.012

