# Supplementary material for: Engineered exosomes with RNA‐motif short hairpin RNA loading and Rab4‐boosted production enable controlled ferroptosis in endometrial carcinoma
Source: Bioeng Transl Med. 2026 Aug 28;11(5):e70163. doi: 10.1002/btm2.70163 (PMC13522897; doi:10.1002/btm2.70163)
Supplement: Supplementary file 1 [file BTM2-11-e70163-s001.docx]

Engineered exosomes with RNA Motif-Enhanced shRNA Loading and Rab4-DrivenAmplified Production for Controlled Ferroptosis Induction in Endometrial Carcinoma

Jiarui Zhang^a,b,1^, Yuwei Yao^a,1^, Wan Shu^a^, Shuangshuang Cheng^a^, Guanglei Zhong^a^, Jia Yu^a^, Jinhua Chen^a^, Kejun Dong^a^, Yingying Peng^a^, Jun Zhang^a^ ^,*^ and Hongbo Wang^a,c*^

*^a^ Department of Obstetrics and Gynecology, Union Hospital, Tongji Medical College, Huazhong University of Science and Technology, Wuhan, 430000, China.*

*^b^ Hubei Key Laboratory of Biological Targeted Therapy, Union Hospital, Tongji Medical College, Huazhong University of Science and Technology, Wuhan, 430000, China.*

*^C^ Hubei Provincial Clinical Medical Research Center for Cancer Immunotherapy, Wuhan, 430000, China.*

*^*^ Corresponding author: 2418009102@qq.com;* *drwanghb69@hust.edu.cn .*

*^1^ These authors contribute equally to this work.*

***Key words: Therapeutic exosomes; Targeted therapy; RNA motif; Endometrial carcinoma; Ferroptosis.***

Figure S1.Validation of the TetR-TetO component. (A)The expression of EGFP in HUVEC cells with/without TCN. (B) Quantitative analysis of fluorescence intensity of EGFP.

Figure S2. (A) The relative expression of EGFP after shGPX4-M vector transfection. (B)The relative expression of EGFP after miR-15a-M vector transfection.

Figure S3. Standard curve for absolute qPCR quantification. The linear regression demonstrates the relationship between the log-transformed synthetic RNA copy numbers (log_10_ (Copy Number)) and the corresponding cycle threshold (Ct) values. The standard curve exhibits an optimal slope of -3.32, corresponding to an amplification efficiency of approximately 100%, with a high correlation coefficient (R^2^ > 0.99).

Figure S4. (A) The relative expression of shGPX4 in ExoM normalized by miR138-5p. (B) Western bolt reveal the expression level of Rab4 in ISK cells after ExoM treatmen

FigureS5. Original uncropped multi-field fluorescence images validating Rab4 knockdown and enhanced exosome retention. Representative wide-field images of ISK cells treated with Exo (top panels) and ExoM (bottom panels). DAPI (blue) indicates nuclei, Rab4 is stained in green, and exosomes are labeled with DiD (red). These images correspond to the quantitative analysis presented in main Figures 4B and 4C.

Figure S6. Colloidal and physiological stability of engineered exosomes. (A) Polydispersity Index (PDI) of ExoM and cRGD-ExoM analyzed by dynamic light scattering. The PDI values below 0.3 indicate highly monodisperse vesicle populations without aggregation. (B) Serum stability profile of cRGD-ExoM. The exosomes were incubated in PBS containing 10% fetal bovine serum (FBS) at 37°C, and the hydrodynamic diameter was monitored over a 48-hour period. Data are presented as mean ± SD (n = 3)

Figure S7.(A,B) Colony formation assay showed the effect of cRGD-ExoM on ISK cells proliferation (****p < 0.0001, ***p<0.001). (C,D) EdU assay showed the effect of cRGD-ExoM on ISK cells proliferation (**p < 0.01, *p<0.05, ns means no significance).

Figure S8.Ratiometric JC-1 assay evaluating the effect of cRGD-ExoM on the mitochondrial membrane potential of ISK cells. Red fluorescence indicates healthy J-aggregates, while green fluorescence indicates depolarized monomers. The bar chart on the right displays the quantitative single-cell ratiometric analysis (Ratio of aggregates to monomers), rigorously normalized to the number of viable cells per field. (****p < 0.0001, ***p<0.001, *p<0.05).

Figure S9. The STR results of ISK cell lines compared to the NICR database.

Figure S10. The validation of HUVEC cells for purity through vWF immunofluorescence.

Figure S11. Structural characterization of the DSPE-PEG2000-cRGDfK conjugate. The 1H-NMR spectrum (in d6-DMSO) confirms the structural integrity and purity of the commercially acquired targeting lipid-peptide conjugate. The dominant peak at ~3.5 ppm corresponds to the repeating ethylene glycol units of the PEG2000 chain, while the peaks in the aliphatic region (0.8-1.5 ppm) and aromatic/amide region (7.0-8.0 ppm) confirm the presence of the DSPE lipid tail and the cRGDfK peptide, respectively.
